# Supplementary material for: Clonality, Mutation and Kaposi Sarcoma: A Systematic Review
Source: Cancers (Basel). 2022 Feb 25;14(5):1201. doi: 10.3390/cancers14051201 (PMC8909603; doi:10.3390/cancers14051201)
Supplement: Supplementary file 1 [file cancers-14-01201-s001.zip › cancers-1513880-SI.pdf]

Supplementary material:

S1: Detailed search strategy for MEDLINE and WEB OF SCIENCE

**MEDLINE via Pubmed**		N references obtained
**1**	Sarcoma, Kaposi [MeSH]	
**2**	Kaposi sarcoma* [Text Word]	
**3**	Clonal Evolution [MeSH]	
**4**	Mutation [MeSH]	
**5**	Polymorphism, Genetic [MeSH]	
**6**	Clone Cells [MeSH]	
**7**	clonal* [Text Word]	
**8**	monoclonal* [Text Word]	
**9**	oligoclonal* [Text Word]	
**10**	polyclonal* [Text Word]	
**11**	Cell Proliferation [MeSH]	
**12**	mutat* [Text Word]	
**13**	reactiv* [Text Word]	
**14**	1 OR 2	1.988
**15**	3 OR 4 OR 5 OR 6 OR 7 OR 8 OR 9 OR 10 OR 11 OR 12 OR 13	90.954
**16**	14 AND 15	2.150
**Final search (performed until 31/09/2020).**		
(kaposi* AND sarcoma* OR Sarcoma, Kaposi[MH]) AND (clonal* OR monoclonal* OR oligoclonal* OR polyclonal* OR mutat* OR reactiv* OR Clonal Evolution[MH] OR Mutation[MH] OR Polymorphism, Genetic[MH] OR Clone Cells[MH] OR Cell Proliferation[MH])		

**WEB OF SCIENCE**		N references obtained
**1**	TS="kaposi sarcoma*"	3.773
**2**	TS="Clonal Evolution"	
**3**	TS=Mutation	
**4**	TS="Genetic Polymorphism"	
**5**	TS="Clone Cells"	
**6**	TS=clonal*	
**7**	TS=monoclonal*	
**8**	TS=oligoclonal*	
**9**	TS=polyclonal*	
**10**	TS="cell Proliferation"	
**11**	TS=mutat*	
**12**	TS=reactiv*	
**13**	2 OR 3 OR 4 OR 5 OR 6 OR 7 OR 8 OR 9 OR 10 OR 11 OR 12 OR 12	2.234.218
**14**	1 AND 13	1.1802
**Final search (performed until 31/09/2020).**		
(((((((((((TS=(clonal*)) OR TS=(monoclonal*)) OR TS=(oligoclonal*)) OR TS=(policlonal*)) OR TS=(polyclonal*)) OR TS=(mutat*)) OR TS=(reactive*)) OR TS=("clonal evolution")) OR TS=(mutation)) OR TS=("genetic polymorphism")) OR TS=("clone cell*")) OR TS=("cell proliferation")) AND (kaposi AND sarcoma)		

S2: Standardised data extraction form

Reviewed by:	
**STUDY DETAILS**	
Study ID/record number	
Study title	
Authors	
Pub. Year	
Country	
Aims of the study	
**STUDY METHOD**	
Study design	
Follow up or study duration	
Sample size	
Sample details	
Context	
Population	
Phenomena of interest: (Clonality7Mutation/…)	
**STUDY RESULTS**	
Description of the investigation(s) or determination(s) used	
Outcomes and it's measurements	
Study method (Design of investigation and statiscal analysis)	
Main results	
**OTHER RELEVANT INFORMATION**	
Funding	
COI disclosure	
Authors comments/discussion	
Reviewer comments	
Notes	
Last update	
